# Supplementary material for: Molecular and circuit mechanisms underlying avoidance of rapid cooling stimuli in C. elegans
Source: Nat Commun. 2024 Jan 5;15:297. doi: 10.1038/s41467-023-44638-5 (PMC10770330; doi:10.1038/s41467-023-44638-5)
Supplement: Supplementary file 1 — Supplementary information [file 41467_2023_44638_MOESM1_ESM.pdf]

Supplemental information for

Molecular and circuit mechanisms underlying avoidance of rapid cooling stimuli in *C. elegans*

Chenxi Lin, Yuxin Shan, Zhongyi Wang, Hui Peng, Rong Li, Pingzhou Wang, Junyan He, Weiwei Shen, Zhengxing Wu, Min Guo*

*Corresponding author email: minguo@mail.hzau.edu.cn

Supplemental information includes:

Supplementary figures 1-19

Supplementary figures

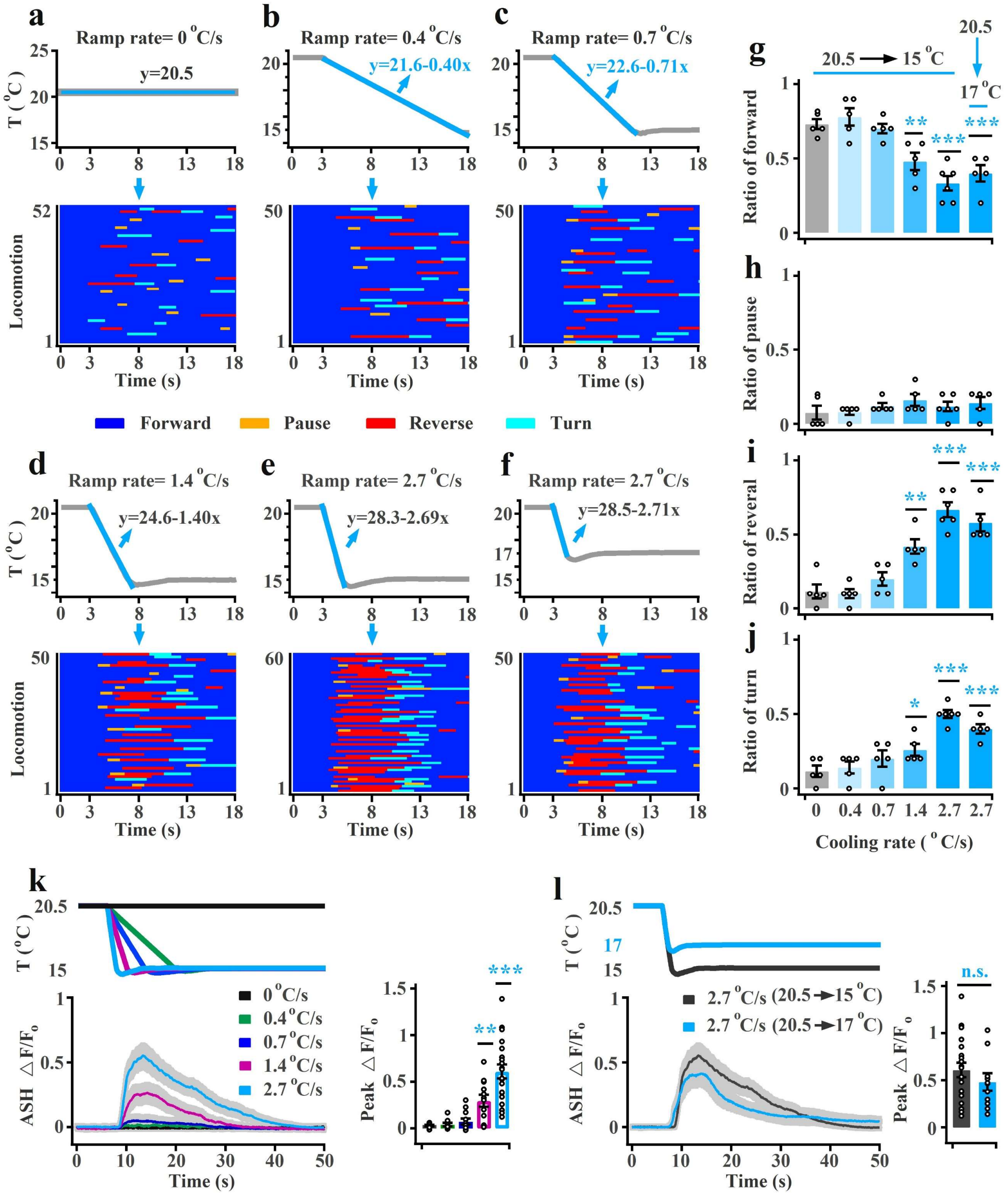

Supplementary figure 1. locomotion behavior of worms and ASH calcium transients elicited by different cooling rates and ranges. **Related to figure 1**.

(**a-f**) Locomotion behavior evoked by different cooling rates and ranges in the forward movement worms. Cooling in the range of 20.5 °C to 15 °C with rate of 0 °C/s (**a**), 0.4 °C/s (**b**), 0.7 °C/s (**c**), 1.4 °C/s (**d**), 2.7 °C/s (**e**), and cooling in the range of 20.5 °C to 17 °C with rate of 2.7 °C/s (**f**). In each figure, upper panel is temperature trace, the light grey line is the average of recording temperature trace. The deep blue line is the fitting curve, and the linear fitting function is on the

right. Lower panel is the worms' locomotion behaviors evoked by the different temperature stimuli. Shown on the left axis is the number of worms. One of four discrete states, including forward (dark blue), pause (yellow), reverse (red) and turn (light blue), is assigned to each trace of worm. (**g-j**) Calculation of the ratio of forward (**g**), pause (**h**), reversal (**i**) and turn (**j**) in figures **a-f**. A reversal or turn is defined by the worm stopping forward movement and initiating a reversal with at least half a head swing or initiating a turn, respectively, within 3-8 seconds in the locomotion map. An omega turn following reversal without interruption was also defined as a turn. n = 5, 5, 5, 5, 6 and 5 groups for each bar in **g-j**, ≥ 10 worms/group. (**k-l**) ASH calcium transients induced by different cooling rates (**k**) and ranges (**l**) in wild-type worms. The different color traces in upper panel indicate different cooling rates (**k**) and ranges (**l**), respectively. The colored lines and the light gray area surrounding them in lower panel indicate the mean values of calcium signal and SEM, respectively. Hereinafter the same shall apply in this manuscript. n = 12, 10, 12, 12 and 20 worms for each bar in **k**. n = 20 and 11 worms for each bar in **l**. All data are expressed as mean ± SEM. One-way ANOVA test followed by Dunnett's multiple comparisons or Kruskal–Wallis test with Dunnett's multiple comparisons in **g-k**. Student's *t test* (two-sided) was performed in **l**. $*p < 0.05$, $**p < 0.01$, $***p < 0.001$, $p > 0.05$ denotes not significant (n.s.).

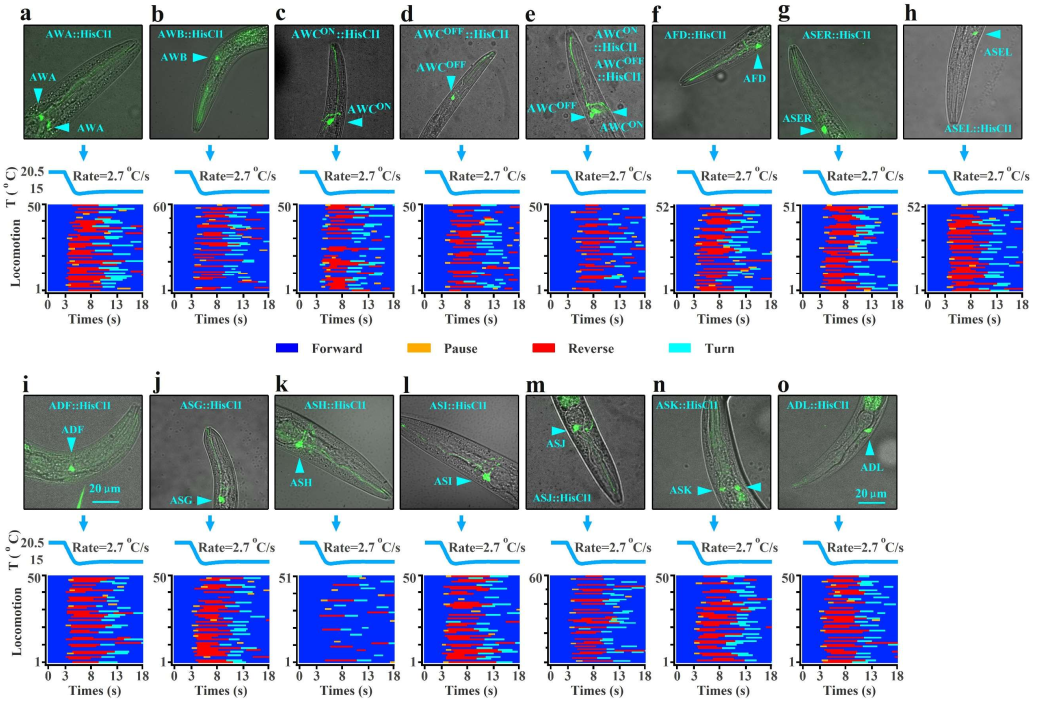

Supplementary figure 2. Recording the rapid cooling-evoked locomotion behavior in sensory neuron-silenced worms. **Related to figure 1**.

(**a-o**) Upper panel: Expression of HisCl1 in the screening sensory neurons, scale bar, 20 µm. Lower panel: Locomotor behavior induced by 2.7 °C/s cooling rate stimulation in the sensory neuron-silenced worms. Silencing neuron activity by expression of HisCl1 plus application of exogenous histamine (HIS). The promotors *odr-7*, *str-1*, *str-2*, *srsx-3*, (*str-2*+*srsx-3*), *gcy-23*, *gcy-5*, *gcy-7*, *srh-142*, *gcy-15*, *sra-6*, *gpa-4*, *trx-1*, *sra-9*, *ver-2* were used to specifically label AWA, AWB, AWC^{ON}, AWC^{OFF}, AWC^{ON+OFF}, AFD, ASER, ASEL, ADF, ASG, ASH, ASI, ASJ, ASK, ADL neurons, respectively. The blue line on the top of locomotion map is temperature trace. Shown on left axis is the number of worms. Hereinafter the same shall apply in this manuscript.

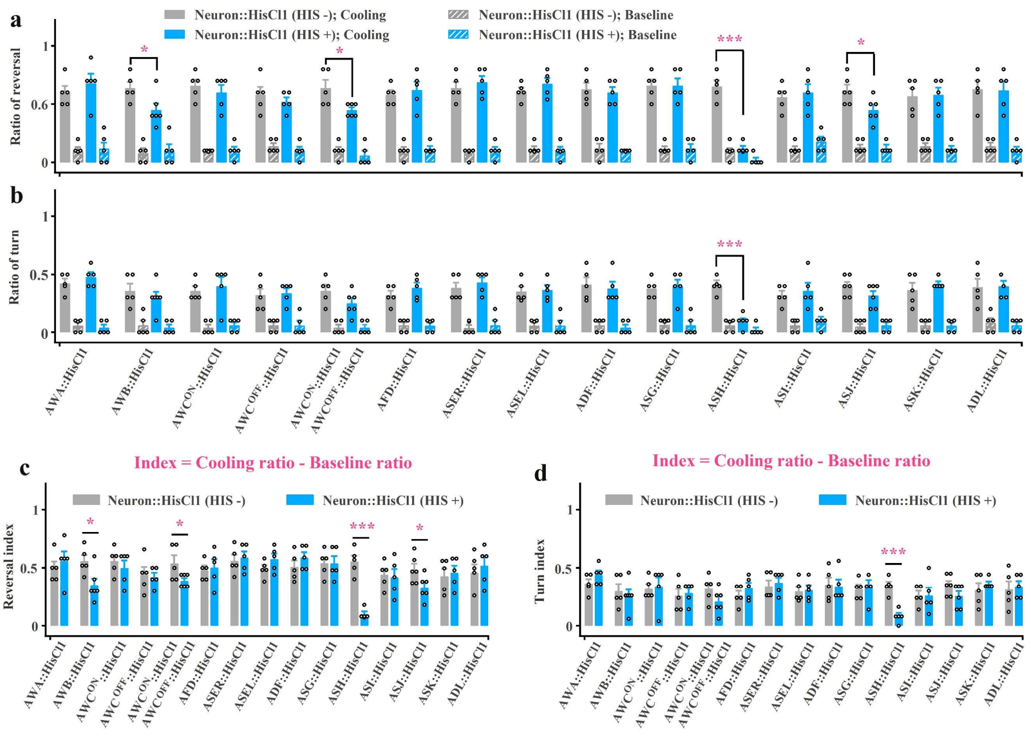

Supplementary figure 3. Screening of candidate sensory neurons that function in rapid cooling-evoked avoidance behavior. **Related to supplementary figure 2 and figure 1**.

(**a-b**) Calculating the ratio of reversal (**a**) and turn (**b**) with or without rapid cooling stimulation in the worms denoted in **Supplementary figure 2**. Cooling-elicited reversal in a forward worm was defined as stopping forward movement and initiating a reversal with at least half a head swing within 5 seconds of stimulation initiation, whereas a cooling-elicited turn was defined as any of the

following: (1) stopping forward movement and initiating a turn within a 5 seconds period and (2) an omega turn following the cooling-evoked reversal without interruption. For baseline reversal or turn definition, the locomotion behaviors at least 8 s in a worm which exhibiting an initiating forward movement without application of cooling stimulation were recorded, and a reversal or turn that occurred within 3-8 s was defined a baseline reversal or baseline turn. In addition, an omega turn following the baseline reversal without interruption was also defined a baseline turn. (**c-d**) Calculation of reversal and turn index induced by rapid cooling in the worms denoted in **Supplementary figure 2**. Reversal or turn index was defined as ratio of reversal or turn with cooling stimulation minus baseline ratio of reversal or turn without cooling, respectively. Hereinafter the same in this manuscript. n = 5, 5, 5, 5, 5, 5, 6, 5, 5, 5, 5, 5, 5, 5, 5, 5, 5, 5, 6, 5, 5, 5, 5, 5, 5, 5, 5, 5, 5, 5, 5, 5, 5, 5, 5, 5, 5, 5, 5, 5, 5, 5, 5, 5, 5, 5, 5, 6, 5, 6, 5, 5, 5, 5, 5, 5, 5, 5 and 5 groups for each bar in **a** or **b**. n = 5, 5, 5, 6, 5, 5, 5, 5, 5, 6, 5, 5, 5, 5, 5, 5, 5, 5, 5, 5, 5, 5, 5, 5, 6, 6, 6, 5, 5, 5and 5 groups for each bar in **c** or **d**, ≥ 10 worms/group. Data are expressed as mean ± SEM. Student's *t test* or Mann–Whitney rank sum tests (two-sided) in figure. **p* < 0.05, ****p* < 0.001.

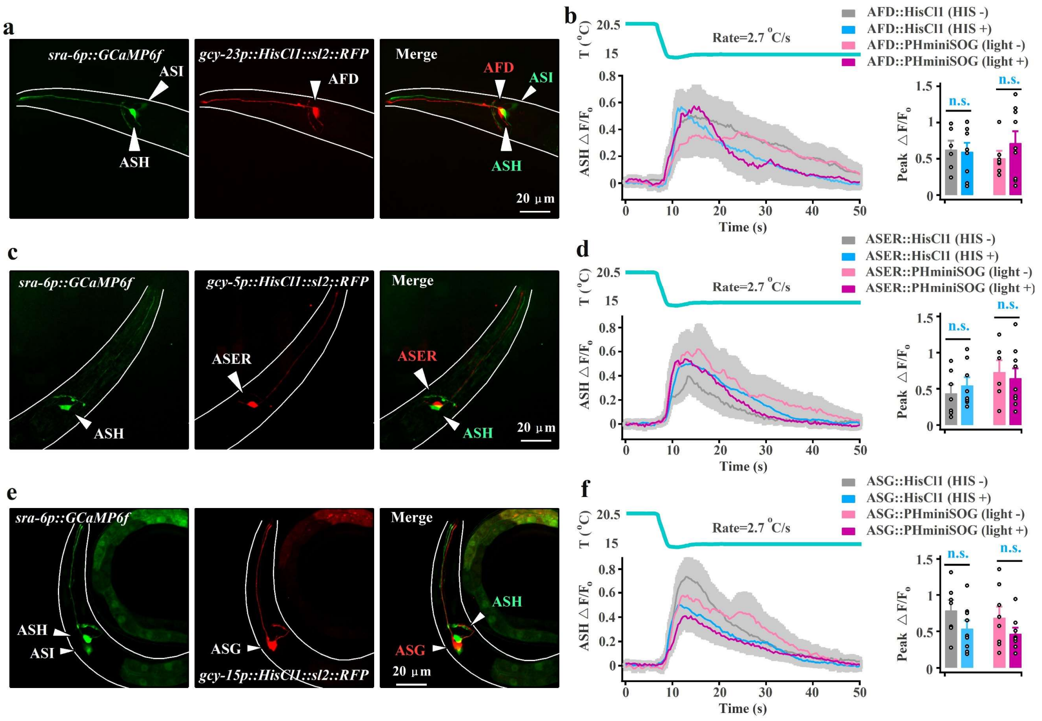

Supplementary figure 4. ASH calcium transients induced by rapid cooling in sensory neurons AFD-, ASER- and ASG-inhibited worms. **Related to figure 2**.

(**a**) The specific expression of GCaMP6f in ASHs, and HisCl1 in AFDs. The *gcy-23* promoter was used to specifically label sensory neurons AFD. Scale bar, 20 μm. (**b**) ASH calcium transients induced by rapid cooling in AFD-inhibited worms. Expressing HisCl1 plus exogenous histamine (HIS) to silence neuron activity, and expressing PHminiSOG plus the periodic illumination of blue light to kill neuron. Hereinafter the same shall apply in this manuscript. n = 6, 9, 7 and 9 worms for each bar. (**c**) The specific expression of GCaMP6f in ASHs, and HisCl1 in ASER. The *gcy-5* promoter was used to specifically label sensory neuron ASER. (**d**) ASH calcium transients induced by rapid cooling in ASER-inhibited worms. n = 7, 8, 6 and 9 worms for each bar. (**e**) The specific expression of GCaMP6f in ASHs, and HisCl1 in ASGs. The promoter of *gcy-15* was used to specifically label sensory neurons ASG. (**f**) ASH calcium transients induced by rapid cooling in ASG-inhibited worms. n = 7, 9, 8 and 8 worms for each bar. Data are expressed as mean ± SEM. Student's *t test* or Mann–Whitney rank sum tests (two-sided) in figure. $p > 0.05$ denotes not significant (n.s.).

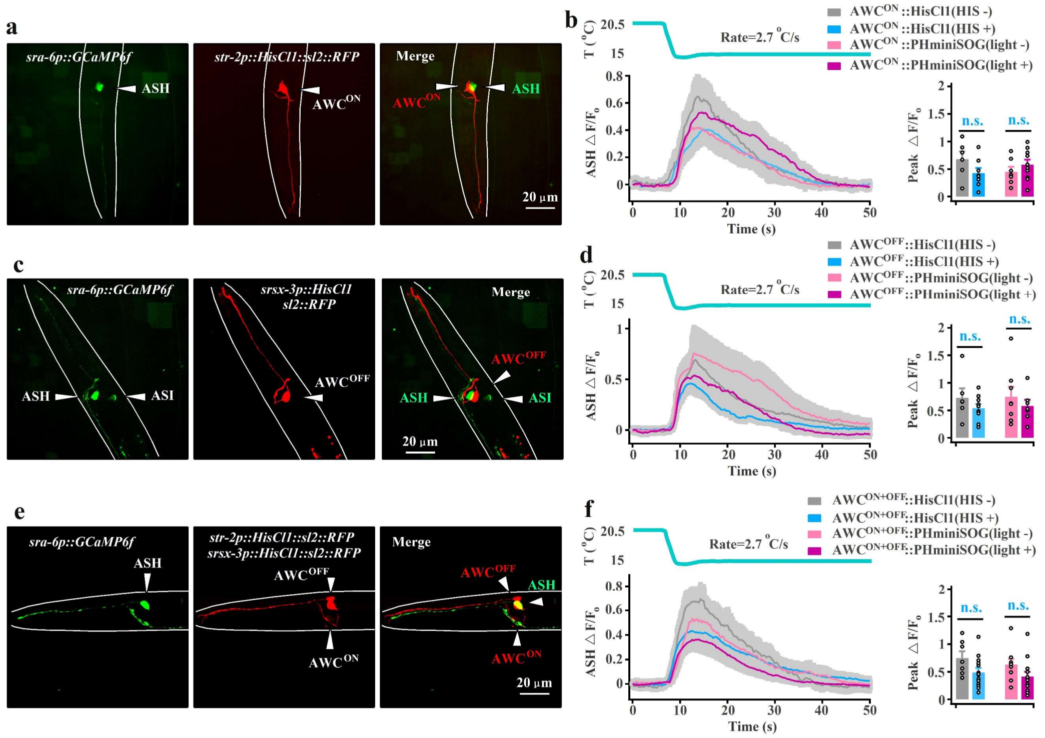

Supplementary figure 5. ASH calcium transients induced by rapid cooling in sensory neurons AWC^{ON}-, AWC^{OFF}- and AWC^{ON+OFF}-inhibited worms. **Related to figure 2**.

(**a**, **c**, **e**) The specific expression of GCaMP6f in ASHs, and HisCl1 in AWC^{ON} (**a**), AWC^{OFF} (**c**) and

AWC^{ON+OFF} (**e**), respectively. The promoters of *str-2*, *srsx-3,* and both *str-2* and *srsx-3* were used to specifically label AWC^{ON}, AWC^{OFF} and AWC^{ON+OFF}, respectively. Scale bar, 20 µm. (**b**, **d**, **f**) ASH calcium transients evoked by rapid cooling in sensory neurons AWC^{ON}- (**b**), AWC^{OFF}- (**d**), and AWC^{ON+OFF}-inhibited (**f**) worms. n = 6, 8, 7 and 10 worms for each bar in **b**. n = 6, 10, 8 and 7 worms for each bar in **d**. n = 7, 14, 9 and 13 worms for each bar in **f**. Data are expressed as mean ± SEM. Student's *t test* or Mann–Whitney rank sum tests (two-sided) in figure. $p > 0.05$ denotes not significant (n.s.).

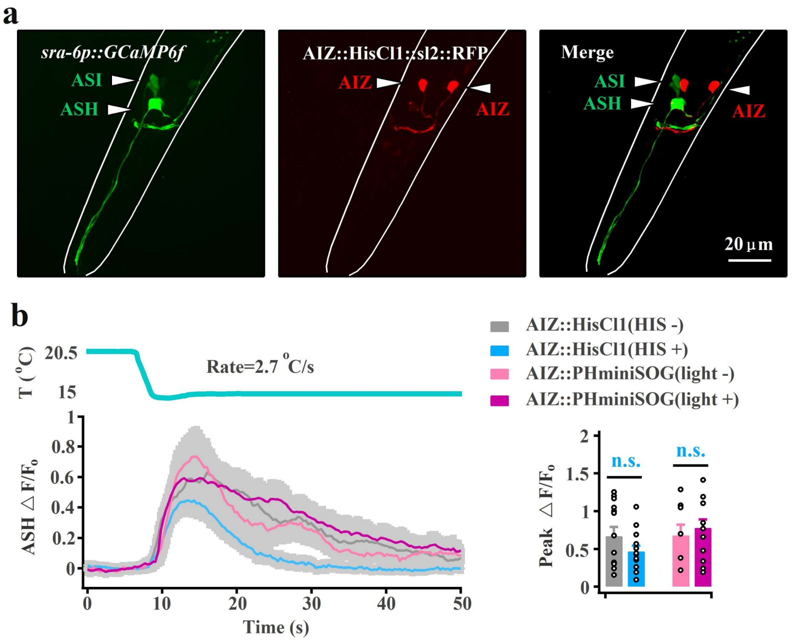

Supplementary figure 6. ASH calcium transients induced by rapid cooling in interneuron AIZ-inhibited worms. **Related to figure 2**.

(**a**) The specific expression of GCaMP6f in ASHs, and HisCl1 in interneurons AIZ. The *ser-2(2)* and *odr-2b* promoters are used to specifically label AIZ. Scale bar, 20 µm. (**b**) ASH calcium transients evoked by rapid cooling in AIZ-inhibited worms. n = 12, 12, 6 and 10 worms for each bar. Data are expressed as mean ± SEM. Student's *t test* or Mann–Whitney rank sum tests (two-sided) in figure. $p > 0.05$ denotes not significant (n.s.).

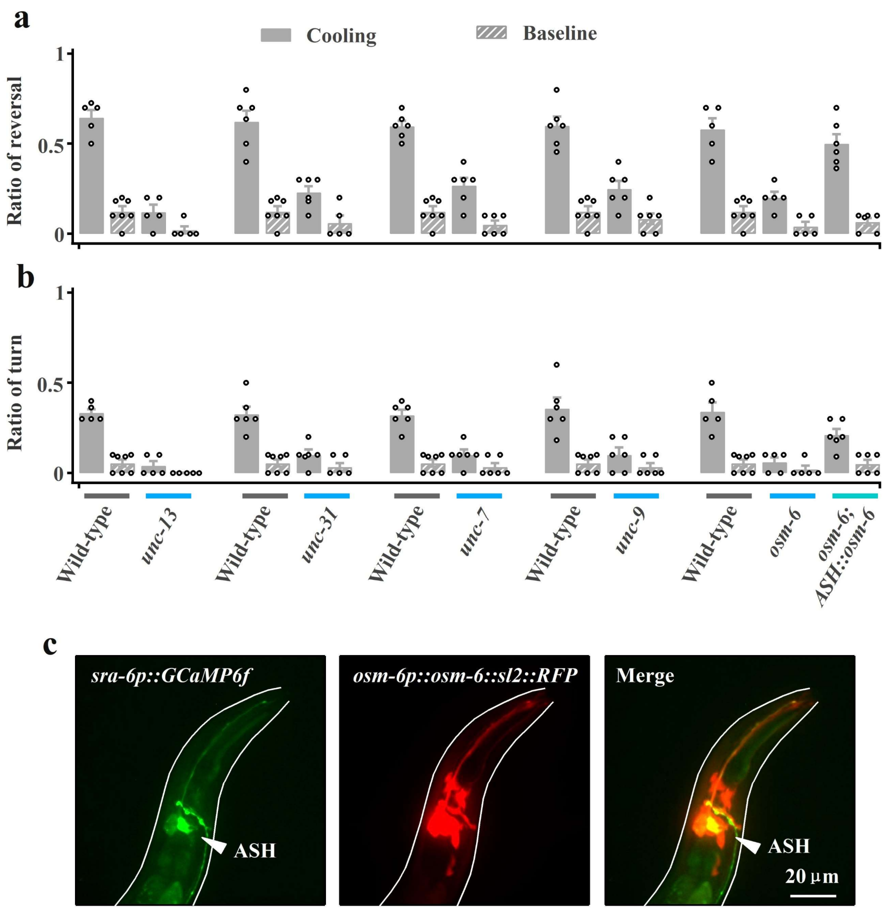

Supplementary figure 7. Calculating the ratio of reversal or turn with or without rapid cooling stimulation in *unc-13, unc-31, unc-7, unc-9, osm-6* mutants, and *osm-6; ASH::osm-6* genetically rescued worms, and the expression pattern of *osm-6*. **Related to figure 2**.

(**a**) Calculating the ratio of reversal. (**b**) Calculating the ratio of turn. n = 5, 7, 5, 5, 6, 7, 6, 5, 6, 7, 6, 6, 6, 7, 6, 6, 5, 7, 5, 5, 6 and 6 groups for each bar in **a** or **b**, and ≥ 10 worms/group. (**c**) Expression pattern of *osm-6p::osm-6::sl2::RFP* and ASH::GCaMP6f in *osm-6* mutant worm, showing OSM-6 expressed in ASH. Scale bar, 20 μm. Data are expressed as mean ± SEM.

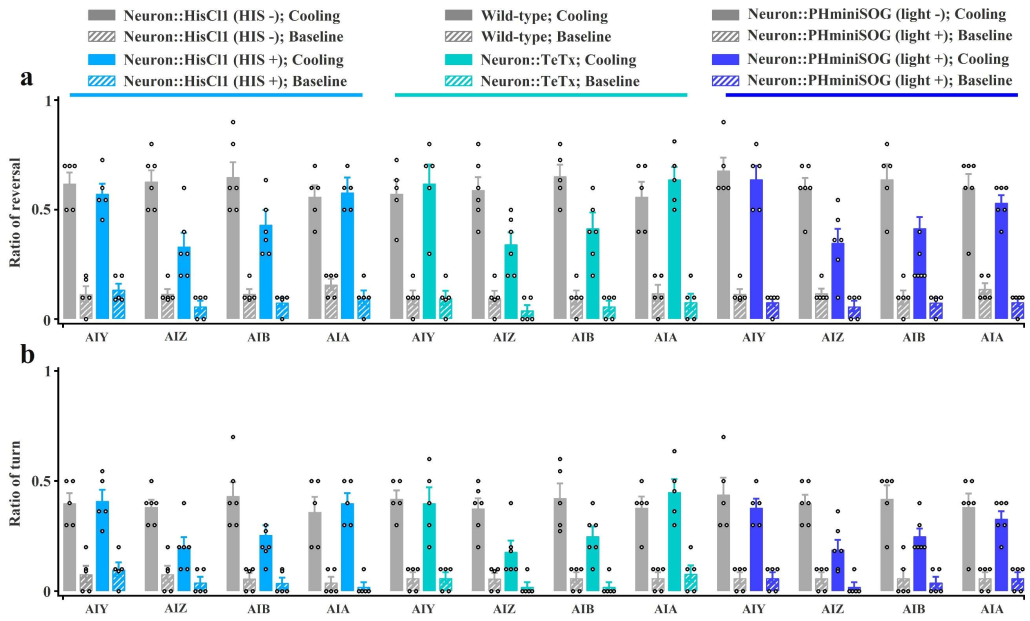

Supplementary figure 8. Calculating the ratio of reversal and turn with or without rapid cooling stimulation in those first-layer interneuron-inhibited worms. **Related to figure 3**.

(**a**) Calculating the ratio of reversal. (**b**) Calculating the ratio of turn. Three methods were used to inhibit the activity of neuron. First, expressing HisCl1 plus exogenous HIS. Second, expressing TeTx. Third, expressing PHminiSOG plus the periodic illumination of blue light. Hereinafter the same shall apply in this manuscript. n = 5, 5, 5, 5, 6, 5, 6, 5, 6, 5, 6, 5, 5, 5, 5, 5, 5, 5, 5, 5, 6, 5, 6, 5, 5, 5, 6, 5, 5, 5, 5, 5, 5, 5, 5, 5, 6, 5, 6, 5, 5, 5, 6, 5, 6, 5, 6 and 5 groups for each bar in **a** or **b**, and ≥ 10 worms/group. Data are expressed as mean ± SEM.

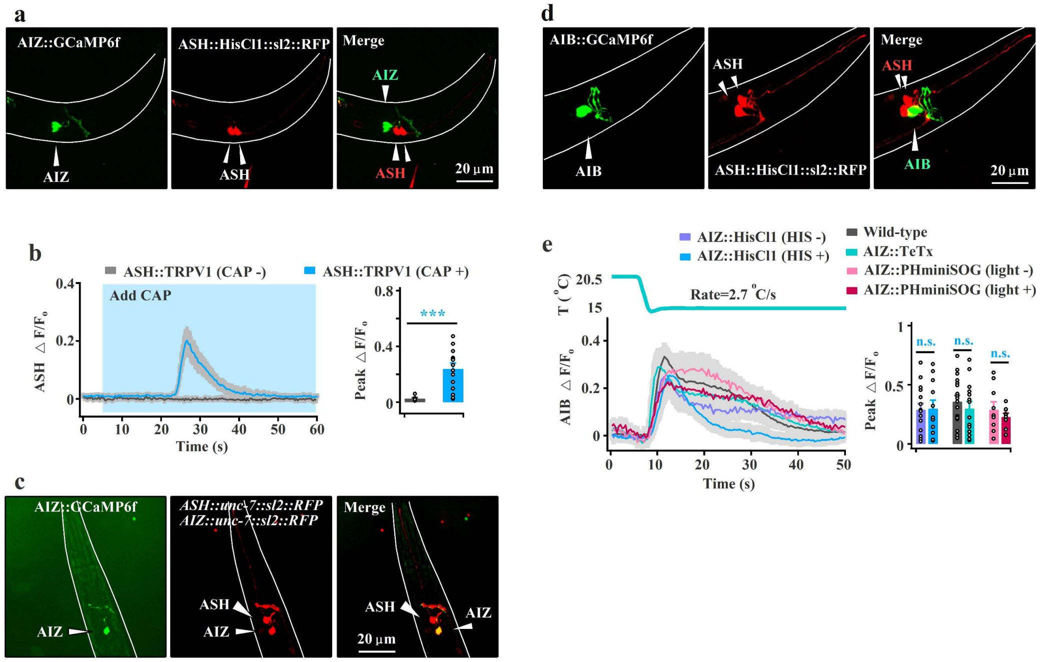

Supplementary figure 9. Expression patterns and calcium activities of neurons in different neuron-manipulated background. **Related to figure 3**.

(**a**) The specific expression of GCaMP6f in AIZ, and HisCl1 in ASH. Scale bar, 20 μm. (**b**) Recording ASH calcium transients elicited by chemogenetic method instead of rapid cooling stimulation. ASHs with expression of TRPV1 were activated by application of exogenous 100 μM capsaicin (CAP). Light blue shading denotes the period of capsaicin application. Hereinafter the same shall apply in this manuscript. n = 9 and 15 worms for each bar. (**c**) The specific expression of GCaMP6f in AIZ, and *unc-7* genomic DNA in both ASH and AIZ neurons. Scale bar, 20 μm. (**d**) The specific expression of GCaMP6f in AIB, and HisCl1 in ASH. (**e**) AIBs calcium transients induced by rapid cooling stimulation in AIZ-inhibited worms. n = 15, 11, 19, 14, 9 and 9 worms for each bar. All data are expressed as mean ± SEM. Student's *t test* or Mann–Whitney rank sum tests (two-sided) in figures **b**, **e**. ***$p < 0.001$, $p > 0.05$ denotes not significant (n.s.).

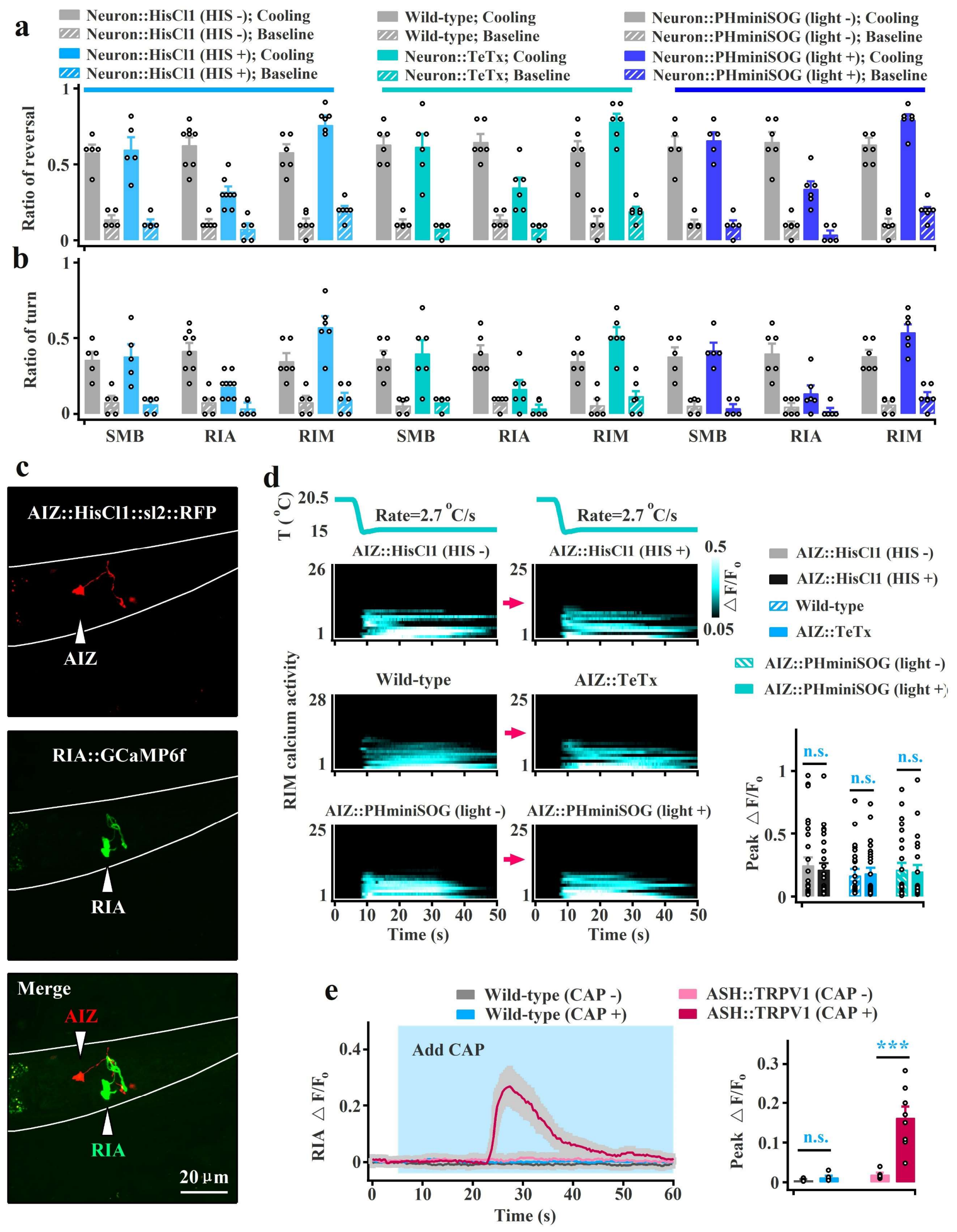

Supplementary figure 10. Examination of locomotion behavior with or without rapid cooling stimulation in those inter/motor neuron-inhibited worms, and testing calcium activity of RIM and RIA in different neuron-manipulated background. **Related to figure 4**.

(**a-b**) Calculating the ratio of reversal (**a**) and turn (**b**) with or without rapid cooling stimulation in those inter/motor neuron-inhibited worms. n = 5, 5, 5, 5, 8, 5, 9, 5, 6, 6, 6, 6, 6, 5, 6, 5, 6, 5, 6, 5, 6, 5, 6, 6, 5, 5, 5, 5, 6, 6, 6, 5, 6, 6, 6 and 7 groups for each bar in **a** or **b**, and ≥ 10 worms/group. (**c**)

The specific expression of HisCl1 in AIZ neurons, and GCaMP6f in RIA neurons. Scale bar, 20 µm. (**d**) Heat maps of RIMs calcium transients elicited by rapid cooling stimuli in the worms of AIZ-silenced, AIZ-blocked, AIZ-killed, respectively. n = 26, 25, 28, 25, 25 and 25 worms for each bar. (**e**) RIA calcium transients following chemogenetic activation of ASHs. n = 7, 8, 7 and 8 worms for each bar. All data are expressed as mean ± SEM. Statistical analysis in **a-b** and **d-e** were performed Student's *t test* or Mann–Whitney rank sum test (two-sided). ***$p < 0.001$, $p > 0.05$ denotes not significant (n.s.).

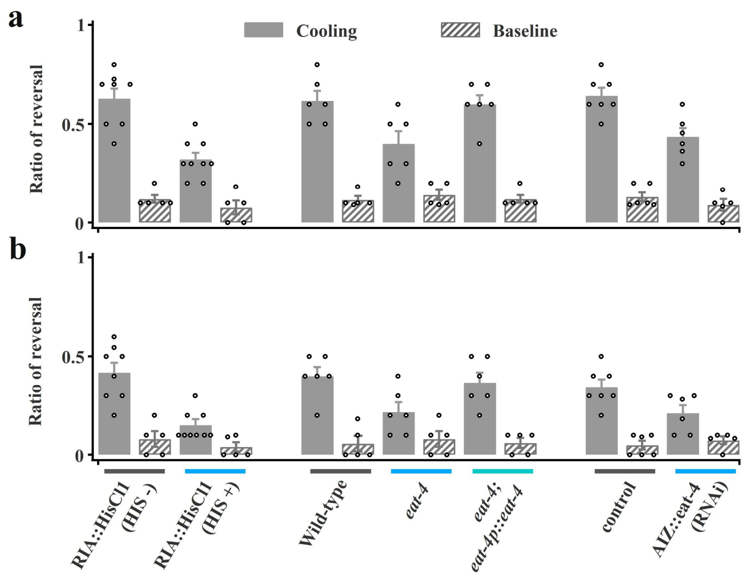

Supplementary figure 11. Calculating the ratio of reversal and turn with or without rapid cooling stimulation in RIA-silenced, *eat-4* mutant, *eat-4; eat-4p::eat-4* and AIZ::*eat-4(RNAi)* worms. **Related to figure 5**.

(**a**) Calculating the ratio of reversal. (**b**) Calculating the ratio of turn. The expression of AIZ::GCaMP6f in wild-type worms were used as the control of AIZ::*eat-4*(RNAi). n = 8, 5, 9, 5, 6, 5, 6, 5, 6, 5, 7, 6, 6 and 5 groups for each bar in **a** or **b**, and ≥ 10 worms/group. All data are expressed as mean ± SEM.

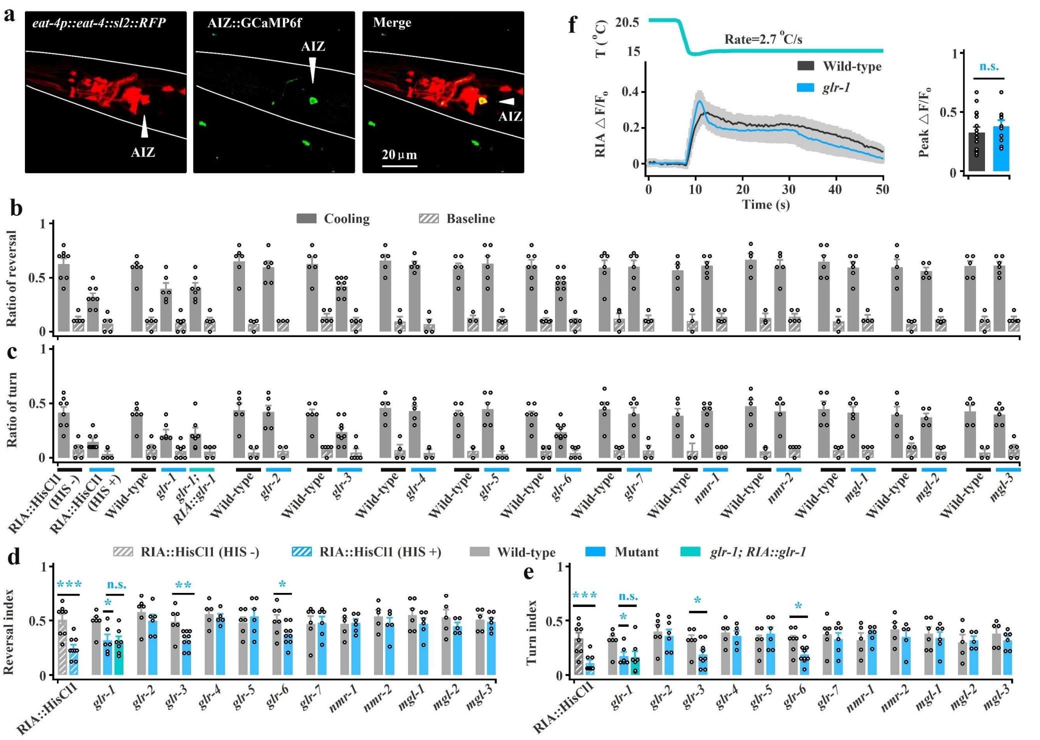

Supplementary figure 12. EAT-4 expression pattern, screening of candidate excitatory glutamate receptors, and comparison of RIA calcium signals between wild-type and *glr-1* mutant worms. **Related to figure 5**.

(**a**) Expression pattern of *eat-4p::eat-4::sl2::RFP* and AIZ::GCaMP6f in *eat-4* mutant worm, showing EAT-4 expressed in AIZ. Scale bar, 20 μm. (**b-c**) Calculating the ratio of reversal (**b**) and turn (**c**) with or without rapid cooling stimulation in glutamate receptor mutant worms. n = 8, 5, 9, 5, 6, 5, 6, 7, 7, 5, 6, 4, 6, 3, 6, 5, 9, 6, 5, 4, 5, 4, 6, 3, 6, 5, 7, 6, 9, 6, 6, 4, 6, 4, 5, 3, 6, 5, 5, 3, 5, 5, 6, 4, 6, 4, 5, 4, 5, 5, 5, 4, 6 and 5 groups for each bar in **b** or **c**, and ≥ 10 worms/group. (**d-e**) Calculation of reversal and turn index induced by rapid cooling in the worms denoted in **b-c**. n = 8, 9, 6, 6, 7, 6, 6, 6, 9, 5, 5, 6, 6, 7, 9, 6, 6, 5, 6, 5, 5, 6, 6, 5, 5, 5 and 6 groups for each bar in **d** or **e**, and ≥ 10 worms/group. (**f**) Comparison of RIA calcium transients induced by rapid cooling stimuli between wild-type and *glr-1* mutant worms. n = 16 and 10 worms for each bar. All data are expressed as mean ± SEM. Student's *t test* or Mann–Whitney rank sum tests (two-sided) in **d-f**. One-way ANOVA test followed by Dunnett's multiple comparisons in **d-e**. *$p < 0.05$, **$p < 0.01$, ***$p < 0.001$, $p > 0.05$ denotes not significant (n.s.).

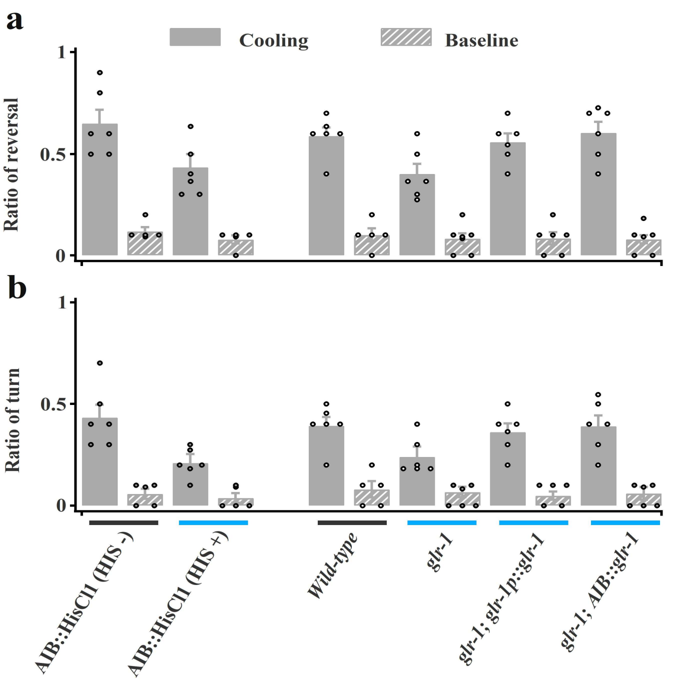

Supplementary figure 13. Calculating the ratio of reversal and turn with or without rapid cooling stimulation in AIB-silenced, *glr-1* mutant, *glr-1; glr-1p::glr-1* and *glr-1; AIB::glr-1* genetically rescued worms. **Related to figure 6**.

(**a**) Calculating the ratio of reversal. (**b**) Calculating the ratio of turn. n = 6, 5, 6, 5, 6, 5, 6, 7, 6, 6, 6 and 6 groups for each bar in **a** or **b**, and ≥ 10 worms/group. Data are expressed as mean ± SEM.

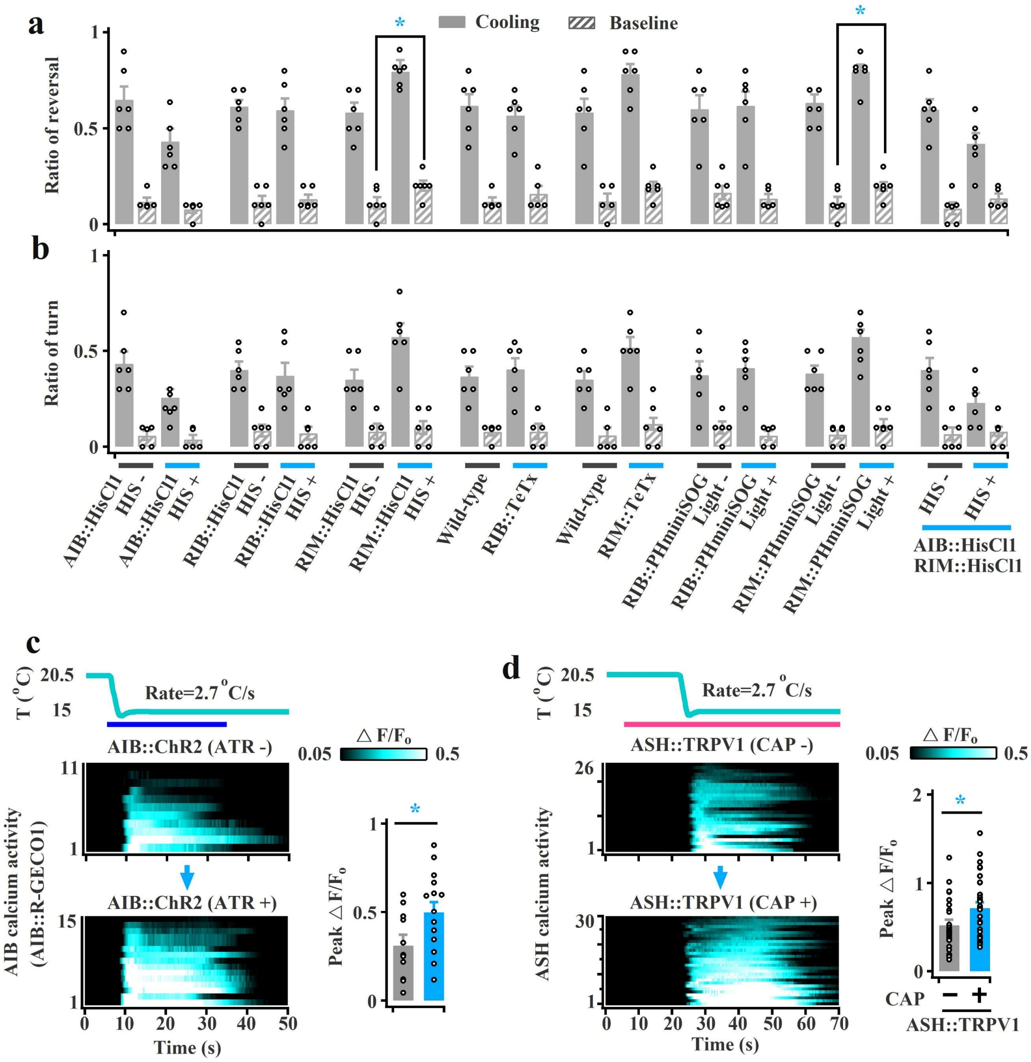

Supplementary figure 14. Examination of locomotion behavior with or without rapid cooling stimulation in those inter/motor neuron-inhibited worms, and testing calcium activities of AIB and ASH in activating themselves through artificial methods during rapid cooling stimulation. **Related to figure 7**.

(**a**) Calculating the ratio of reversal. (**b**) Calculating the ratio of turn. n = 6, 5, 6, 5, 6, 6, 6, 6, 6, 6, 6, 6, 6, 5, 6, 5, 6, 5, 6, 6, 6, 6, 6, 5, 6, 6, 6, 6, 6, 6 and 5 groups for each bar in **a** or **b**, and ≥ 10 worms/group. (**c**) Heat maps of AIB calcium response to the rapid cooling in optogenetic activation of AIB worms. The genetically encoded Ca^{2+} sensor R-GECO1 was expressed in AIB to test the calcium signal. AIB that specifically expressed ChR2 were activated by blue light illumination plus 5 μM All-Trans-Retinal (ATR). The dark blue line indicates the period of blue light illumination. n = 11 and 15 worms for each bar. (**d**) Heat maps of ASH calcium response to the rapid cooling in

chemogenetic activation of ASH worms. For both rapid cooling and capsaicin stimulation, capsaicin was delivered to the worm nose 17 seconds before cooling by using a programmable automatic drug feeding device. The red line indicates the period of capsaicin application. n = 26 and 30 worms for each bar. Data are expressed as mean ± SEM. Student's *t test* or Mann–Whitney rank sum test (two-sided) in figure (*$p < 0.05$).

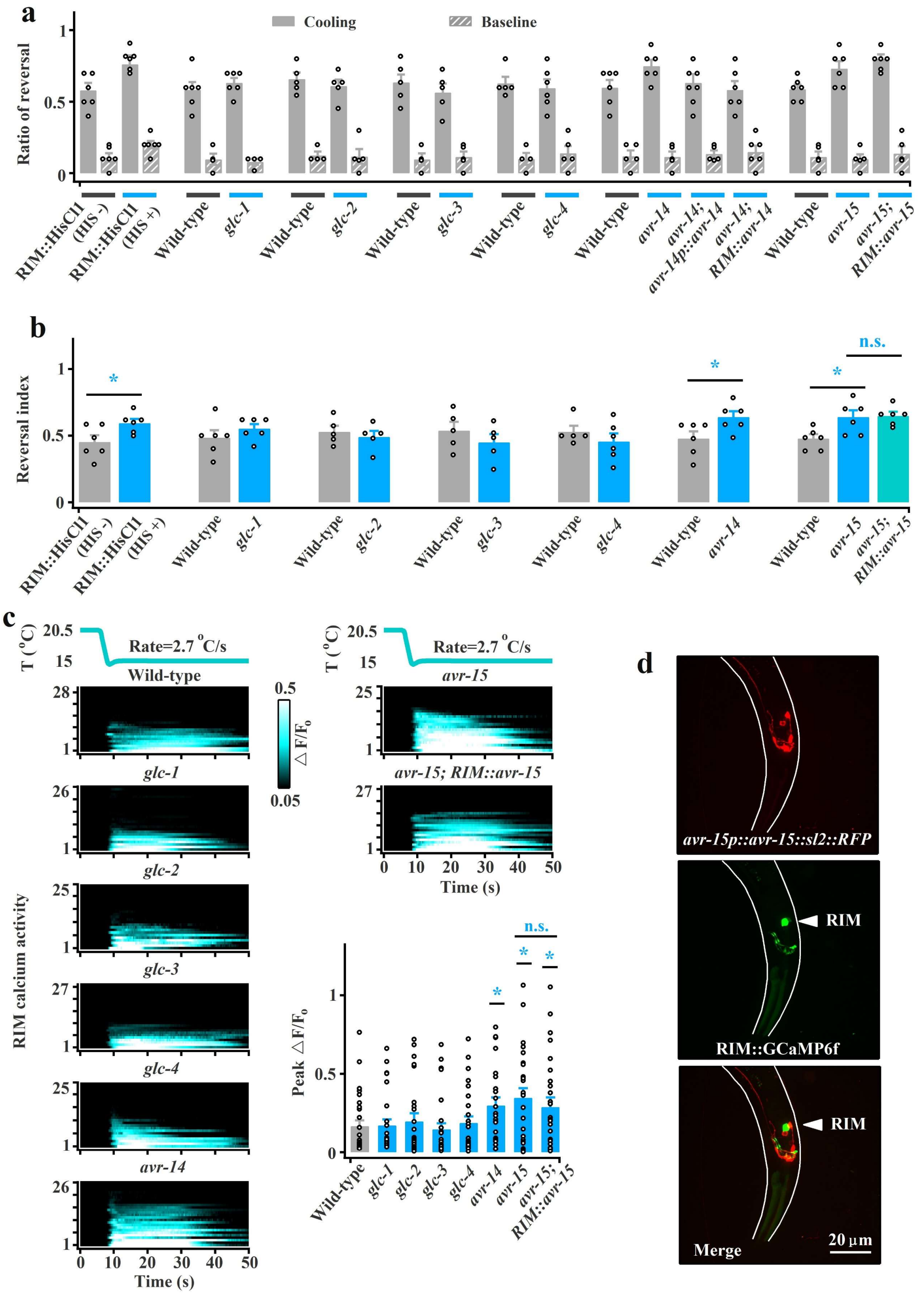

Supplementary figure 15. Screening of candidate glutamate-gated chloride channels that function in inhibition of RIM activities, and expression pattern of *avr-15p::avr-15::sl2::RFP*. **Related to figure 8.**

(**a**) Calculating the ratio of reversal with or without rapid cooling stimulation in glutamate-gated

chloride receptor null mutant worms. n = 6, 6, 6, 6, 6, 4, 6, 4, 5, 4, 5, 5, 5, 4, 5, 5, 5, 4, 6, 5, 6, 5, 6, 5, 7, 5, 6, 6, 6, 5, 6, 5, 6 and 5 groups for each bar, and ≥ 10 worms/group. (**b**) Calculating the reversal index in the worms denoted in **a**. n = 6, 6, 6, 6, 5, 5, 5, 5, 5, 6, 6, 6, 7, 6, 6, 6 and 6 groups for each bar, and ≥ 10 worms/group. (**c**) Heat maps of RIM calcium response to rapid cooling stimuli in the worms denoted in **a-b**. n = 28, 26, 25, 27, 25, 26, 25 and 27 worms for each bar. (**d**) Expression pattern of *avr-15p::avr-15::sl2::RFP* and RIM::GCaMP6f in *avr-15* mutant, showing AVR-15 is not expressed in RIMs. Scale bar, 20 µm. All data are expressed as mean ± SEM. Student's *t test* or Mann–Whitney rank sum test (two-sided) was performed in **b**. Kruskal–Wallis test with Dunnett's multiple comparisons in **c**. *$p < 0.05$, $p > 0.05$ denotes not significant (n.s.).

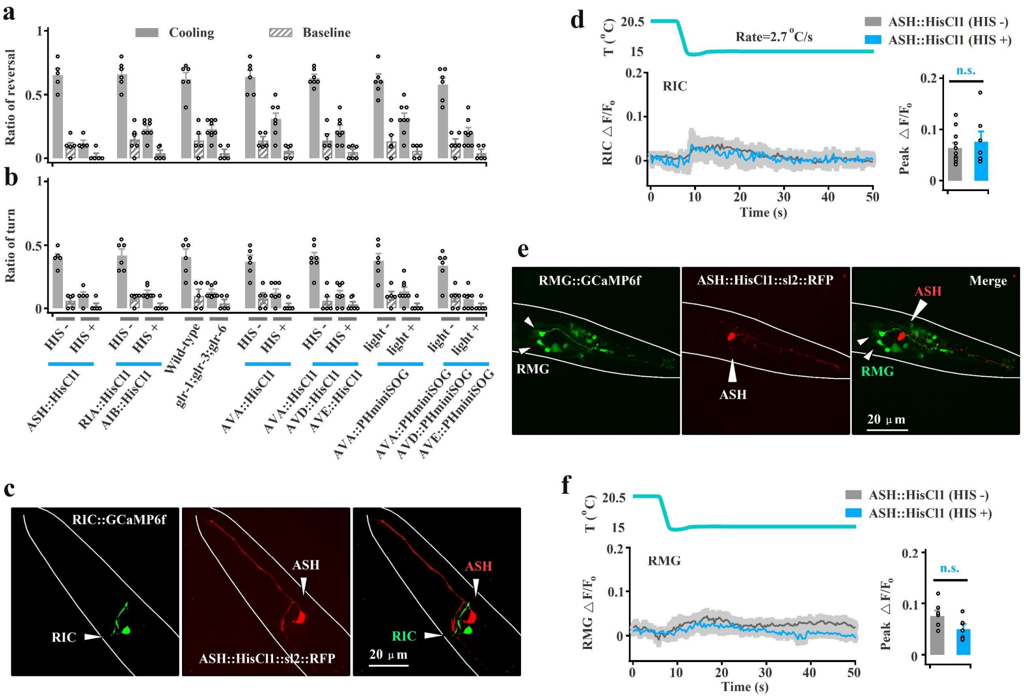

Supplementary figure 16. Calculating the ratio of reversal and turn with or without rapid cooling stimulation in those interneuron-inhibited worms, and testing the cooling-evoked calcium activities of interneurons RIC and RMG in ASH-inhibited worms. **Related to figure 9**.

(**a**) Calculating the ratio of reversal. (**b**) Calculating the ratio of turn. n = 5, 5, 5, 5, 6, 6, 8, 5, 6, 5, 9, 5, 6, 5, 8, 5, 7, 5, 8, 6, 6, 5, 8, 5, 6, 6, 8 and 5 groups for each bar in **a** or **b**, and ≥ 10 worms/group. (**c**) The specific expression of HisCl1 in ASH neurons, and GCaMP6f in RIC neurons. The *tbh-1*

promoter was used to specifically label interneuron RIC. Scale bar, 20 μm. (**d**) RIC calcium transients induced by rapid cooling in ASH-silenced worms. n = 11 and 7 worms for each bar. (**e**) The specific expression of HisCl1 in ASH neurons, and GCaMP6f in RMG neurons. The *ncs-1* promoter was used to specifically label interneuron RMG. Scale bar, 20 μm. (**f**) RMG calcium transients induced by rapid cooling in ASH-silenced worms. n = 7 worms. All data are expressed as mean ± SEM. Student's *t test* or Mann–Whitney rank sum test (two-sided) was performed in **d, f**. $p > 0.05$ denotes not significant (n.s.).

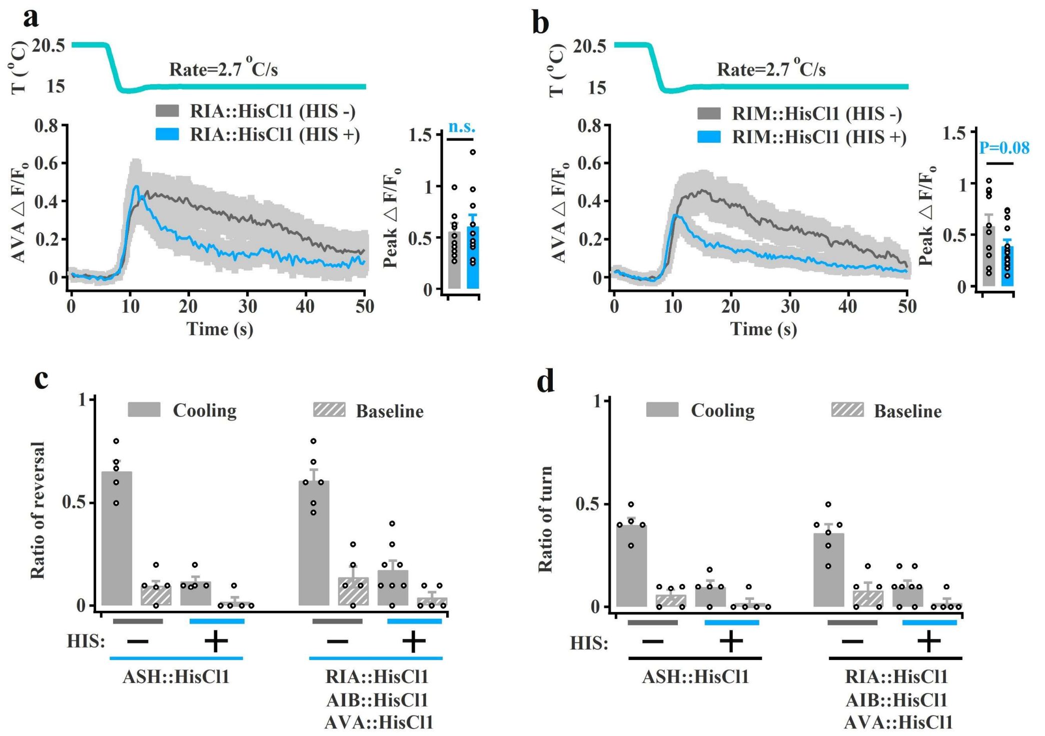

Supplementary figure 17. Recording the cooling-evoked calcium activities of command interneurons AVA after inhibition of RIA or RIM, and calculating the ratio of reversal and turn with or without rapid cooling stimulation in those interneuron-inhibited worms. **Related to figure 10.**

(**a**) AVA calcium transients induced by rapid cooling in RIA-silenced (**a**) and RIM-silenced (**b**) worms. For each bar, n = 11 and 10 worms in figure **a**, and n = 10 and 14 worms in figure **b**. (**c-d**) Calculating the ratio of reversal and turn with or without cooling. n = 5, 5, 5, 5, 6, 5, 8 and 5 groups for each bar in **c** or **d**, and ≥ 10 worms/group. Data are expressed as mean ± SEM. Student's *t test* or Mann–Whitney rank sum test (two-sided) were performed in **c-d**. $p > 0.05$ denotes not significant (n.s.).

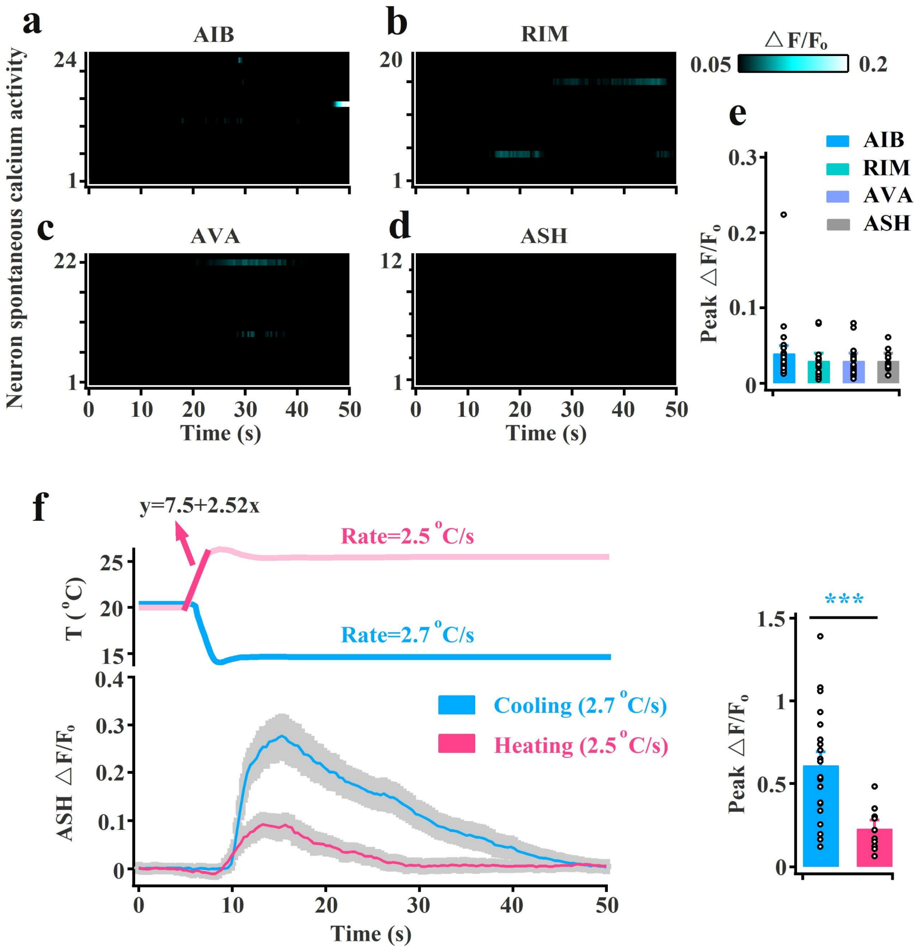

Supplementary figure 18. Recording the spontaneous calcium activities in AIB, RIM, AVA and ASH neurons, and comparing ASH calcium transients induced by rapid heating or rapid cooling in wild-type worms.

(**a-d**) Heatmaps of AIB, RIM, AVA and ASH spontaneous calcium activities that without application of cooling stimulation. (**e**) Comparison of the peak of calcium signal change in the worms denoted in **a-d**. n = 24, 20, 22 and 12 worms for each bar. (**f**) Testing the rapid heating- or cooling-evoked ASH calcium transients in wild-type worms. The rapid heating rate is ~2.5 °C/s, and the rapid cooling rate is ~2.7 °C/s. n = 20 and 13 worms for each bar. Data are expressed as mean ± SEM. Mann–Whitney rank sum test (two-sided) was used in **f**. ***$p < 0.001$.

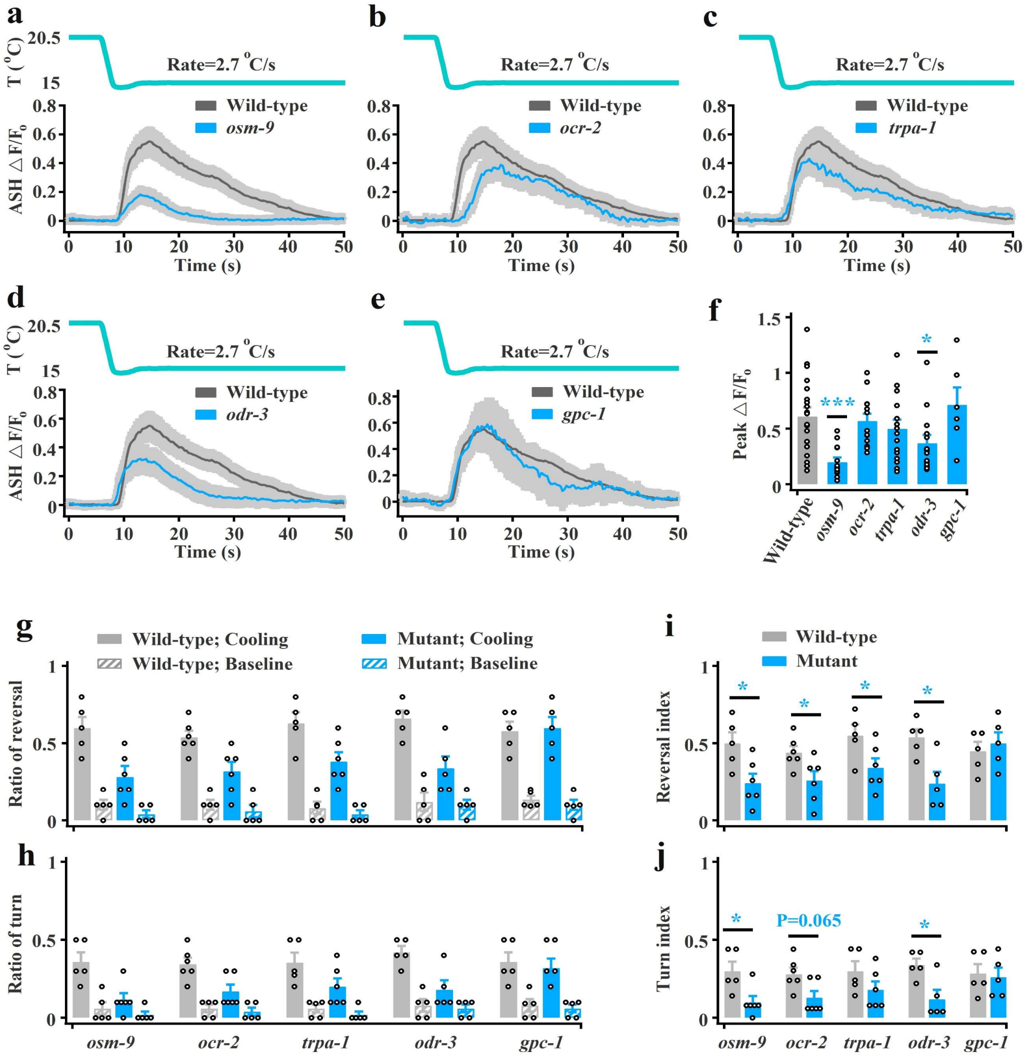

Supplementary figure 19. Screening of candidate sensors that function in ASH to respond to rapid cooling.

(**a-f**) Examination of ASH calcium transients induced by rapid cooling in *osm-9*, *ocr-2*, *trpa-1*, *odr-3*, *gpc-1* mutant worms. n = 20, 14, 13, 15, 14 and 6 worms for each bar in figure **f**. (**g-h**) Calculating the ratio of reversal and turn with or without rapid cooling stimulation in the worms denoted in **a-e**. n = 5, 5, 6, 5, 6, 5, 6, 5, 5, 5, 6, 5, 5, 5, 5, 5, 5, 5, 5 and 5 groups for each bar in **g** or **h**, and ≥ 10 worms/group. (**i-j**) Calculating the reversal and turn index in worms denoted in **a-e**. n = 5, 6, 6, 6, 5, 6, 5, 5, 5 and 5 groups for each bar, and ≥ 10 worms/group. Data are expressed as mean ± SEM. Student's *t test* or Mann–Whitney rank sum test (two-sided) were performed in **i-j**. One-way ANOVA test followed by Dunnett's multiple comparisons in **f**. *$p < 0.05$, ***$p < 0.001$.
