# Supplementary material for: Molecular and circuit mechanisms underlying avoidance of rapid cooling stimuli in C. elegans
Source: Nat Commun. 2024 Jan 5;15:297. doi: 10.1038/s41467-023-44638-5 (PMC10770330; doi:10.1038/s41467-023-44638-5)
Supplement: Supplementary file 3 — Inventory of Additional Supplementary Information [file 41467_2023_44638_MOESM3_ESM.pdf]

Supplementary movie legends

Supplementary movie 1: A wild-type day 1 worm with forward sinusoidal locomotion exhibited no response when its head suffered slowing cooling rate (0.4 °C/s) stimulation. The cooling range is from 20.5 °C to 15 °C. The slow cooling elicits slow bubble atomizing in the movie. **Related to Fig. s1b**.

Supplementary movie 2: A wild-type day 1 worm with forward sinusoidal locomotion exhibited continuous sinusoidal backward locomotion when its head suffered rapid cooling (2.7 °C/s) stimulation. The cooling range is from 20.5 °C to 15 °C. The fast cooling can elicit rapid bubble atomizing in the movie. **Related to Fig. 1c**, **Fig. s1e.**

Supplementary movie 3: ASH neurons in wild-type worm displayed no calcium response to slowing cooling rate (0.4 °C/s) stimulation with cooling range from 20.5 °C to 15 °C. **Related to Fig. s1k.**

Supplementary movie 4: ASH neurons in wild-type worm displayed obvious calcium transients during rapid cooling stimulation. The rapid cooling rate is 2.7 °C/s and cooling range from 20.5 °C to 15 °C. **Related to Fig. 1m, o.**

Supplementary movie 5: Recording the calcium activity in cell body and axon of AIZ neuron during rapid cooling stimulation. The intensity of calcium activity in cell body is stronger than in axon. The rapid cooling rate is 2.7 °C/s and cooling range is from 20.5 °C to 15 °C. **Related to Fig. 3d.**

Supplementary movie 6: Recording the calcium activity in cell body and axon of RIA neuron during rapid cooling stimulation. The cell body and axon of RIA neuron showed similar calcium activity during rapid cooling stimulation. The rapid cooling rate is 2.7 °C/s and cooling range from 20.5 °C to 15 °C. **Related to Fig. 4d.**
